# From HIV protein sequences to viral fitness landscapes: a new paradigm for in silico vaccine design

**DOI:** 10.1186/1742-4690-9-S2-P331

**Published:** 2012-09-13

**Authors:** AL Ferguson, AK Chakraborty

**Affiliations:** 1 University of Illinois at Urbana-Champaign, Urbana, IL, USA; 2 MIT, Cambridge, MA, USA

## Background

An inexpensive prophylactic vaccine offers the best hope to curb the HIV/AIDS epidemic gripping sub-Saharan Africa. Systematic means to guide the design of an effective immunogen for this, and other, infectious diseases are not available. What is required is a method to chart the peaks and valleys of viral fitness as a function of amino acid sequence. An efficacious vaccine would eject the virus from the high fitness peaks, and drive it into the valleys where its compromised fitness impairs its ability to replicate and inflict damage to the host.

## Methods

Appealing to spin glass models in statistical physics, we present a novel approach to translate viral sequence databases into landscapes of viral fitness. These inferred models furnish a quantitative description of viral replicative capacity as a function of amino acid sequence. We illustrate this approach in the development of landscapes for the proteins of HIV-1 clade B Gag.

## Results

In comparisons to experimental and clinical data, our inferred landscapes demonstrate excellent agreement with: 1) in vitro replicative fitness measurements, 2) clinically observed high-fitness circulating viral strains, 3) documented HLA associated CTL escape mutations, and 4) intra-host temporal adaptation pathways revealed by deep sequencing. These favorable comparisons support our landscapes as reflections of intrinsic viral fitness. We illustrate the value of such descriptions in the computational design of a CTL Gag immunogen.

## Conclusion

We present a novel methodology to translate viral sequence data into quantitative landscapes of viral fitness. In an application to HIV-1 Gag, we illustrate excellent agreement of our model predictions with experimental and clinical data, and demonstrate a powerful new approach for HIV immunogen design. We anticipate that this approach may represent a heretofore unprecedented means to synthesize fitness landscapes for diverse pathogens, and may provide the basis for the design of improved prophylactic and therapeutic strategies.

